# A Bibliometric Analysis of hypertension and anxiety from 2004 to 2022

**DOI:** 10.1097/MD.0000000000041859

**Published:** 2025-03-28

**Authors:** Si-Qi Liu, Xin-Yu Ji, Hai-Yi Liang, Shu-Han Zhao, Fu-Yi Yang, Yang Tang, Shuai Shi

**Affiliations:** a Guang’anmen Hospital, China Academy of Chinese Medical Sciences, Beijing, China; b Beijing University of Chinese Medicine Third Affiliated Hospital, Beijing, China; c Institute of Basic Research in Clinical Medicine, China Academy of Chinese Medical Sciences, Beijing, China; d School of Traditional Chinese Medicine, Beijing University of Chinese Medicine, Beijing, China.

**Keywords:** anxiety, bibliometrics, hypertension, risk factors, web of science

## Abstract

**Background:**

A growing body of clinical evidence points to an association between hypertension and anxiety, but the mechanisms by which the two occur are unclear. This article aims to explore possible common influences and associations between hypertension and anxiety.

**Methods:**

We searched for publications on hypertension and anxiety from January 01, 2004 to December 31, 2022 in Web of Science and performed bibliometrics using CiteSpace, VOSviewer, Scimago Graphica and Gephi.

**Results:**

A total of 3216 related articles were retrieved from the Web of Science database. After screening, 3051 articles were included. The number of published articles has increased over the past 19 years. The United States has more researches in this area and has strong collaborative relationships with other countries, which gives it some credibility and authority. The words that appear in the burst keywords are gender, age, obesity, depression, panic disorder, pregnancy induced hypertension, coronary heart disease, chronic kidney disease, and pituitary adrenal axi, which are co-related with hypertension and anxiety.

**Conclusion:**

There is a link between hypertension and anxiety, and the 2 influence each other, usually in a positive way. Common influences on hypertension and anxiety include age, gender, obesity, depression, panic attacks, pregnancy, coronary heart disease and chronic kidney disease. Recent research hotspots have focused on population aging and comorbidities. Future research hotspots are likely continue to focus on influencing factors, clinical research and prognosis.

## 1. Introduction

Hypertension and anxiety are common health problems in the current society, which bring some degree of negative impact on both individuals and society.

Hypertension is a common chronic disease, which can lead to many serious complications, such as heart attack, coronary heart disease (CHD), cerebral infarction, hypertensive nephropathy, retinopathy, etc., which seriously affects the quality of life and longevity of patients. Some studies have predicted that about 115.3 million adults in the United States will suffer from hypertension from 2017 to 2020.^[1]^ The WORLD HEART REPORT 2023 published by the World Heart Federation suggests that 20.5 million people die of cardiovascular disease in 2021, with elevated blood pressure being the main cause of cardiovascular.^[2]^ Numerous clinical studies have shown that there is an association between hypertension and anxiety.^[3–5]^ First of all, hypertensive patients are often prone to anxiety due to the physical discomfort that occurs as a result of pathological changes and the negative emotions about the disease after diagnosis. This anxiety not only exacerbates the patient’s psychological burden, but may also further affect the patient’s blood pressure level and exacerbate the occurrence of hypertension. Second, anxiety itself is an independent risk factor for the development of hypertension, and people who are chronically anxious are more likely to experience abnormal fluctuations in blood pressure than those who are psychologically healthy.

Anxiety is a common psychological disorder that manifests itself in persistent inner turmoil, worry, and fear, and in severe cases can lead to problems such as insomnia, loss of appetite, and poor concentration. According to American Psychological Association, approximately 15 to 20 per cent of people are affected by anxiety disorder at some point in their lives.^[6]^

Anxiety seriously affects people’s quality of life and may further lead to depression, suicidal tendencies and other negative emotions, so the treatment of anxiety disorders also needs to be emphasized. Although a large amount of relevant literature supports the existence of an association between the two, the common influencing factors and occurrence pathways between the two are still unclear.

The aim of this thesis is to conduct further research on the relationship between hypertension and anxiety by visualizing and analyzing the literature related to the two, in order to explore the possible mechanisms of mutual influence between the two. We will review the relevant literature in Web of Science (WOS), synthesize and analyze the existing results through visualization studies, and summarize the existing evidence on the association between hypertension and anxiety. By delving into the relationship between hypertension and anxiety, we can better understand the interplay mechanism between the two and provide reference for clinical practice.

## 2. Materials and methods

Science mapping is an essential procedure of bibliometrics. It can represent the discipline situation and development status. We use CiteSpace, VOSviewer, Scimago Graphica, and Gephi to make visualization mapping in this paper. We conducted a comprehensive search of relevant literature data in WOS using the following search strategy: TS=((Hypertension) AND (Anxiety)) from January 1, 2004, to December 31, 2022. The language of the documents was not limited. In order to avoid the error caused by the technical update of the website, all searches and screening were completed in March 1, 2024. In the document types, article and review article were be chosen to ensure the quality of the paper. Two people screened and checked the literature back to back, and a third person checked the controversial places. The search was conducted with the option “All records and references cited” and exported as “download_XX.txt” files. By reading titles and abstracts, we removed articles that were clearly irrelevant to the topic and finally exported 3216 relevant literature records from WOS for bibliometric and visual analysis. We imported txt into Citespace at the location of the “input” in “data.” Through systematic screening and transformation, 3051 articles were finally exported from the “output.” In this study, OriginPro 2024b(10.1.5.132)^[7]^ was used to chart the number of publications per year. CiteSpace(6.1.R6)^[8]^ and VOSviewer (version 1.6.17)^[9]^ are used as a tool to perform the co-citation analysis to identify research hotspots and predict development trends. CiteSpace can be used to perform basic analyses of literature, such as analyzing the number of citations and publications, key journals, author analysis, research and collaborations in institutions, clustering and bursting keyword analysis. In this paper, we mainly apply Citespace (6.1.R6) software for journal analysis, cluster analysis and bursting keyword analysis. The co-citation analysis of the literature shows the number of publications in scientific journals, the intensity of collaborations and the main year of publication. Cluster analysis clusters the keywords by an algorithm to get the keywords represented by each cluster and assigns a label to each cluster. The more keywords in the clusters, the more significant the cluster structure is represented. Selecting LLR clustering method and clicking “Find Clusters” to explore the common themes of similar documents can help us discover the hidden patterns and regularities in a large amount of text data. Click “Burstness” in the control panel to extract and detect burst terms, and click “Refresh” to calculate the number of bursting keywords. Adjust the Y value to get the suitable number of burst terms, and select the top 25 to be analyzed, in order to understand the frontiers of research, the shift of research focus and the latest research hotspot dynamics, and to help predict the subsequent development trend of the field.

VOSviewer is used to analyze institutions, countries, journals and keywords. The results were shown in different forms, including network visualization, overlay visualization and density visualization to reflect different emphases. Scimago Graphica^[10]^ and Gephi^[11]^ are used for mapping of country co-operation and keyword time zones.

In the clustering maps made by Citespace, cluster colors indicate time. The darker the color, the further back in time the study was conducted; the lighter the color, the more recent the study was. National cooperation relationship diagram and density visualization map are the same.

## 3. Results

### 3.1. Published trend

**Figure 1.**
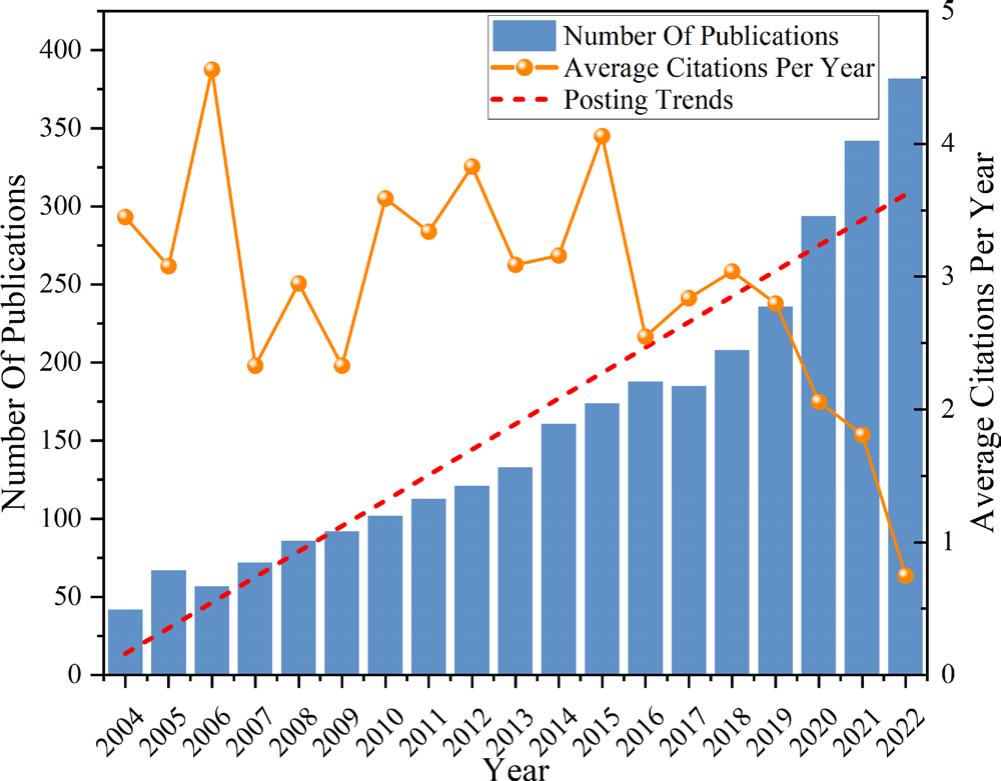
Total number and average citations per year of published literature on hypertension and anxiety.

Figure 1 displays the publishing trend from 2004 (42) to 2022 (382), the number of published papers generally showed a continuous upward trend, reflecting the increasing academic activity of hypertension and anxiety. Research on this field has gained a certain scale and has attracted more attention from scholars. According to the posting trends, we predict that the number of publications in the next few years will also show an upward trend. We can note that the average citations per year in the last 20 years were unstable and showed a fluctuating trend. However, the average citations per year in the last 5 years has been declining which may be related to factors such as the increasingly strict review of papers and the changing national cooperation relationship. The average citations per year is likely to remain low in the coming years. On the whole, the number of papers published in the next few years will still show an upward trend, but the citation will be low. This indicates that the field is receiving increasing attention, but the quality of articles may be uneven.

### 3.2. Author co-citation analysis

#### 3.2.1. Quantity of published articles of authors

As in Table 1, we analyze the 9 authors with the highest number of publications in the field of hypertension and anxiety from the data exported by WOS. The top 2 authors in order of the number of publications are Rihmer Zoltanzhu ya (10) and Rafael R.G. Oganov (10). Rihmer Zoltanzhu ya focuses on psychiatry, neurology, pharmacology, psychology, and cardiovascular system. The research are about depression and anxiety in different hypertensive phenotypes,^[12]^ the relationship between hypertension and emotional temperament^[13–16]^ and cardiovascular abnormalities.^[15,16]^ Rafael R.G. Oganov specializes in the cardiovascular system, biochemistry and molecular biology and pharmacology. He focuses on cardiac and psychosocial risk factors^[17,18]^ in patients with common cardiovascular diseases such as hypertension, prediction of fatal and non-fatal events in arterial hypertension through depression and anxiety.^[19,20]^

**Table 1:** The top 9 prolific authors of hypertension and anxiety research

Author	Documents	Organization	50 h-index
Rihmer Zoltan	10	Semmelweis University Clinical Center	45
Oganov, Rafael R.G.	10	National Research Center for Preventive Medicine of the Ministry of Healthcare of the Russian Federation	19
Nemcsik, Janos	9	Semmelweis University	9
McManus, Richard J.	9	University of Oxford Nuffield Dept Primary Care Hlth Sci	1
Pedersen, Susanne S	9	Tilburg University	46
Kivimaki, Mika	9	University of Helsinki	15
McIntyre, Roger	9	University of Silesia	2
Laszlo, Andrea	9	Norisana MVZ Rosenau	8
Kao, Chia-Hung	9	China Medical University Hospital	44

#### 3.2.2. Analysis of the author’s institution

**Figure 2.**
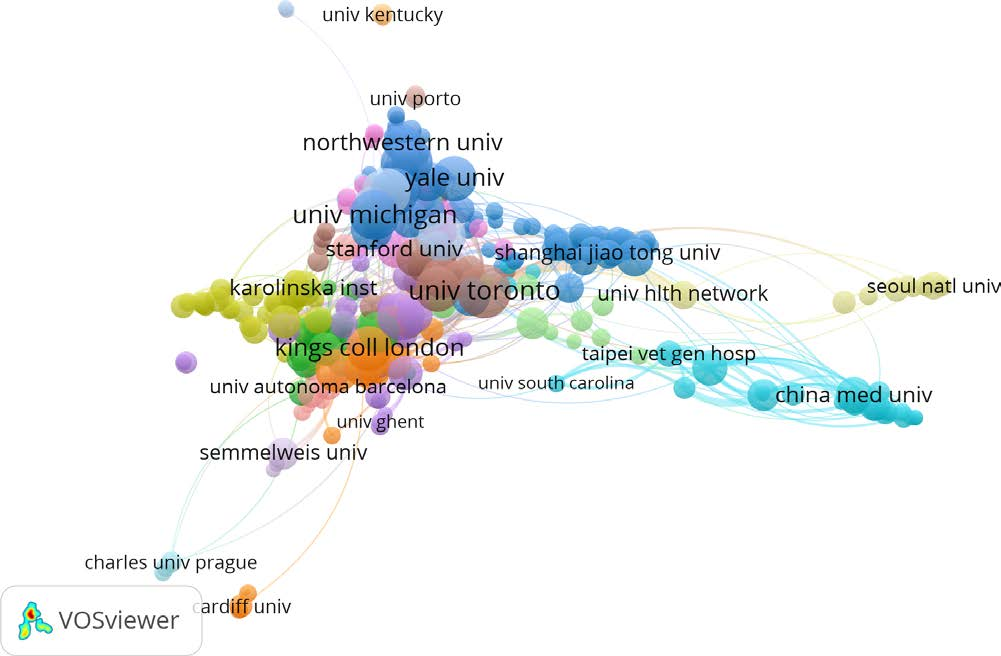
The analysis of institutions. Different colors represent different clusters. The size of the circle is proportional to the number of publications.

The partnerships and publications of different institutions are shown in Figure 2. According to the size of the circle, University of Toronto, King’s College London and Michigan State University have published more papers and cooperated more closely with other institutions. The sample used in this study contains 4536 institutions from 113 countries. The number of publications and average citations of each institution reflect the influence of them. Table 2 shows the top 10 organizations in this field. University of Toronto in the United States ranked 1 with 48 publications and 1796 citations. Michigan State University ranked 2 with 41 publications and 986 citations. Harvard University in the United States ranked 3 with 40 publications and 3099 citations.

**Table 2:** Top 10 influential institutions in hypertension and anxiety research

Organizations	Documents	Citations	Country
University of Toronto	48	1796	Canada
Michigan State University	41	986	United States
Harvard University	40	3099	United States
Harvard med sch	38	677	United States
The University of Melbourne	37	879	Australia
University of San Francisco	37	2202	United States
King’s College London	35	664	United Kingdom
McGill University	35	1363	Canada
Yale university	35	997	United States
University College London	32	2064	United Kingdom

#### 3.2.3. Analysis of the author’s country

**Figure 3.**
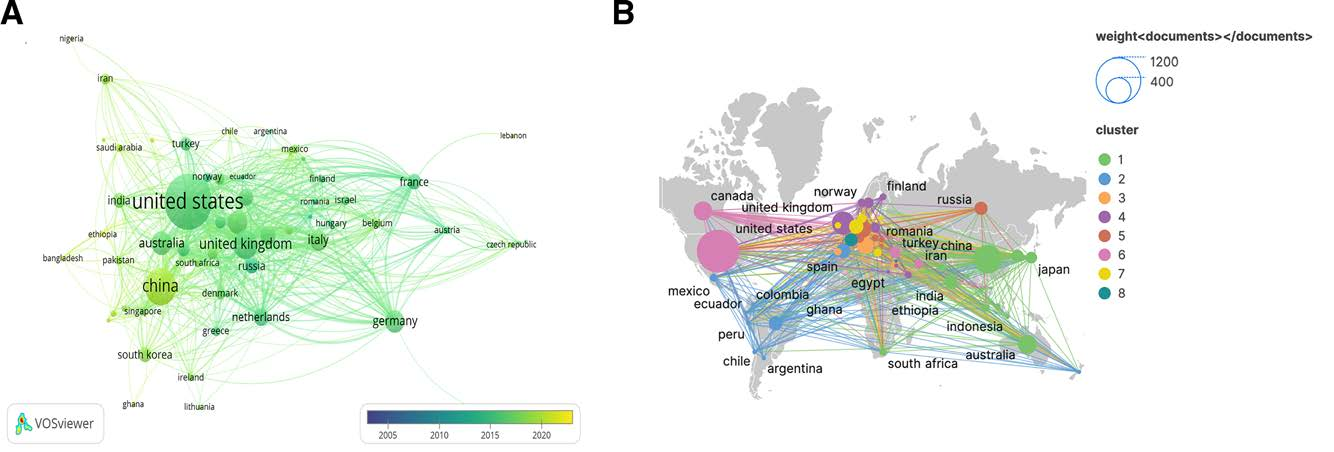
The analysis of countries. (A) National Cooperation Relationship Diagram. (B) Network map of countries engaged in hypertension and anxiety depression research.

It can be found from Figure 3A that the publication years of most countries are mainly concentrated in 2015. The corresponding circle color of China is lighter, indicating that relevant studies in China have gradually increased in recent years. The corresponding circles in the United States and the United Kingdom are larger in area and darker in color, indicating that the research in these countries started earlier and the research content is more abundant.

Figure 3B is a map of relations and cooperation between countries. It can be seen that the United States, as the country with the largest number of articles and citations in this field, has the most complex cooperation relationship, followed by the United Kingdom. Most countries have cooperative relations, which shows that the research in this field is relatively sufficient, and countries exchange and learn from each other to promote development

Combine the data shows in Table 3, the United States is the most impact country with 997 documents and 36,887 citations, which indicating the articles published in the United States have a certain degree of authority. China and United Kingdom are ranked 2 and 3 with 447 documents and 4622 citations, 288 documents and 12,837 citations. Although China has a large number of articles, the number of citations is far lower than that of the United States and the United Kingdom.

**Table 3:** Top 10 countries in terms of publications

Country	Documents	Citations	Total link strength
United States	997	36,887	486
China	447	4622	177
United Kingdom	288	12,837	403
Australia	202	4927	207
Canada	192	7365	160
Germany	181	4562	199
Italy	150	3780	227
Spain	115	2916	172
Netherlands	114	4469	182
Brazil	107	1938	120

### 3.3. Document co-citation analysis

#### 3.3.1. Journal analyses

**Figure 4.**
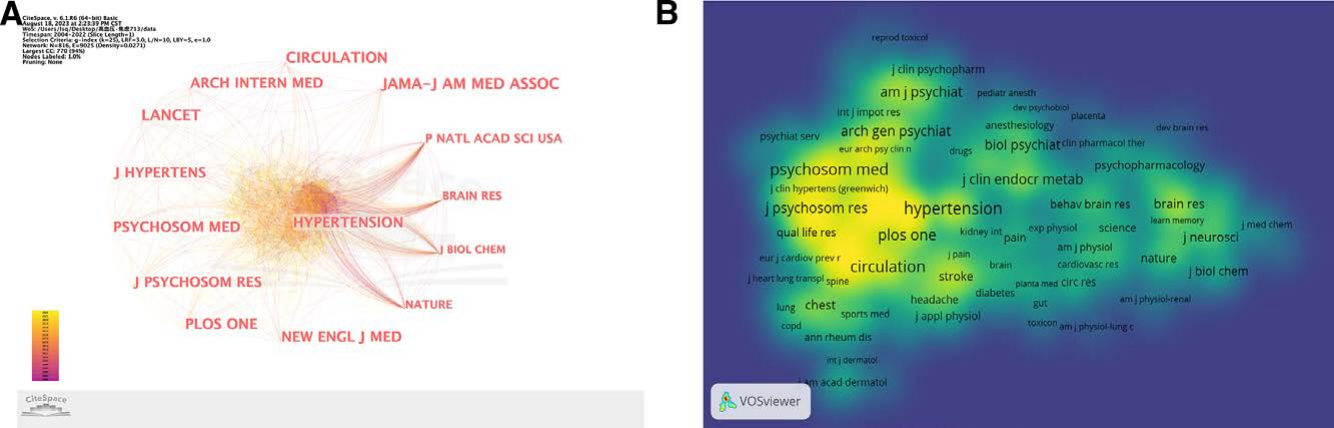
The analysis of journals. (A) Network map of journals related to hypertension and anxiety depression research. (B) The density visualization map of journals related to hypertension and anxiety research. The higher the density, the closer it is to red and the lower the density, the closer it is to blue.

We analyzed the journals and the results are shown in Figure 4. The journals with the most publications are showed in Figure 4A. Combined with the data in Table 4, it can be found that most of the literature on hypertension and anxiety has been published in internal medicine journals. The top 3 most prolific journals were Hypertension (1855), Lancet (1772) and Circulation (1750). The journals that publish the most work closely with other journals. Among the countries to which the journal belongs, 8 are the United States, and the rest are the United Kingdom and the Netherlands. It can be seen that the United States is relatively authoritative in relevant aspects and has a high degree of recognition and conviction. Figure 4B is the density view of the journal. Hypertension, Psychosomatic Medicine, Journal of Psychosomatic Research, Circulation and Plos One have a high density by color. Many articles in related fields are published in these journals.

**Table 4:** The top 10 journals of origin of articles on hypertension and anxiety research

Journal	Element	Country	Citations	Total link strength
Hypertension	Peripheral vascular disease	United States	1855	101,370
Lancet	Medicine, general & internal	England	1772	100,644
Circulation	Cardiac & cardiovascular systems	United States	1750	104,318
Jama-Journal of the American Medical Association	Medicine, general & internal	United States	1676	100,403
Psychosomatic Medicine	Psychiatry, psychology, multidisciplinary psychology	United States	1651	73,605
Journal of Hypertension	Peripheral vascular disease	United States	1455	66,330
Plos One	Multidisciplinary sciences	United States	1420	74,802
The New England Journal of Medicine	Medicine, general & internal	United States	1310	88,006
Journal of Affective Disorders	Clinical neurology, psychiatry	Netherlands	1166	59,661
Archives of General Psychiatry	Psychiatry	United States	1001	60,297

#### 3.3.2. Highly cited literature

**Figure 5.**
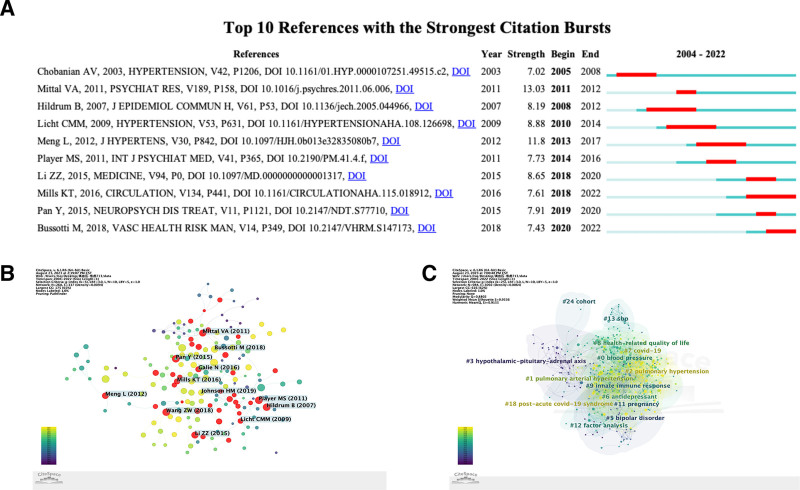
The analysis of co-cited literature. (A) Top 10 references with strongest citation bursts of hypertension and anxiety research. (B) Network map of author analysis of highly cited literature. (C) Cluster analysis of highly cited literature about hypertension and anxiety research.

Figure 5A shows the literature with a large number of citations. Through author analysis (Fig. 5B) and cluster analysis (Fig. 5C) of relevant highly cited literature, authors with greater influence and hot spots in this field have been showed. Combined with the data from Citespace’s automated analysis as in Table 5, the most cited papers were Diagnostic and statistical manual of mental disorders by Mittal VA (30) and Depression increases the risk of hypertension incidence: a meta-analysis of prospective cohort studies (28).

**Table 5:** Top 10 author analysis diagram for highly cited literature

Number	Title	First author	Journal	Citation	Year	Strength
1	Seventh Report of the Joint National Committee on Prevention, Detection, Evaluation, and Treatment of High Blood Pressure	Chobanian AV	Hypertension	12	2003	7.02
2	Diagnostic and statistical manual of mental disorders	Mittal VA	Psychiatry Research	30	2011	13.0
3	Association of low blood pressure with anxiety and depression: the Nord-Trøndelag Health Study	Hildrum B	Journal of epidemiology and community health	17	2007	8.19
4	Depression is associated with decreased blood pressure, but antidepressant use increases the risk for hypertension	Licht CMM	Hypertension	20	2009	8.88
5	Depression increases the risk of hypertension incidence: a meta-analysis of prospective cohort studies	Meng L	Hypertension	28	2012	11.8
6	Anxiety Disorders, Hypertension, and Cardiovascular Risk: A Review	Player MS	The International Journal of Psychiatry in Medicine	19	2011	7.73
7	Prevalence of Depression in Patients with Hypertension A Systematic Review and Meta-Analysis	Li ZZ	Medicine	17	2015	8.65
8	Global Disparities of Hypertension Prevalence and Control: A Systematic Analysis of Population-Based Studies From 90 Countries	Mills KT	Circulation	19	2016	7.61
9	Association between anxiety and hypertension: a systematic review and meta-analysis of epidemiological studies	Pan Y	Neuropsychiatric Disease and Treatment	19	2015	7.91
10	Anxiety and depression in patients with pulmonary hypertension: impact and management challenges	Bussotti M	Vascular Health and Risk Management	19	2018	7.43

After excluding the literature related to pulmonary hypertension, the most cited literature within the field of research on hypertension and anxiety were literature 5 (28) and 4 (20). Literature 5 examined the relationship between depression and hypertension, and through meta-analysis, the researchers concluded that depression is an independent risk factor for hypertension. Literature 4 explored the effect of anxiety, depressive disorders and use of antidepressants on blood pressure and showed that the average diastolic blood pressure was higher in the anxious population, depression was associated with lower systolic blood pressure as well as, to a lesser extent, hypertension, whereas the use of certain antidepressant medications was associated with higher diastolic and systolic blood pressure as well as hypertension.

10 years ago, the cited studies were mainly related to “blood pressure,” “hypothalamic-pituitary-adrenal axis,” “bipolar disorder,” “pregnancy,” “innate immune response,” “factor analysis,” “13 sbp,” “cohort.” In recent years, there has been a shift in research priorities, focusing on “pulmonary hypertension,” “18 post-acute covid-19 syndrome,” “covid-19,” “cohort,” “pulmonary arterial hypertension,” “antidepression,” and “health-related quality of life” (from most recent to most distant in time). Cluster labels were retained for only a fraction of the higher degree of association. The 3 largest clusters are “#0blood pressure,” “#1pulmonary arterial hypertension,” “#2 pulmonary hypertension.” 1 and 2 are both pulmonary hypertension and can be combined, suggesting that the result is related to the search term “ hypertension.”

### 3.4. Keyword

**Figure 6.**
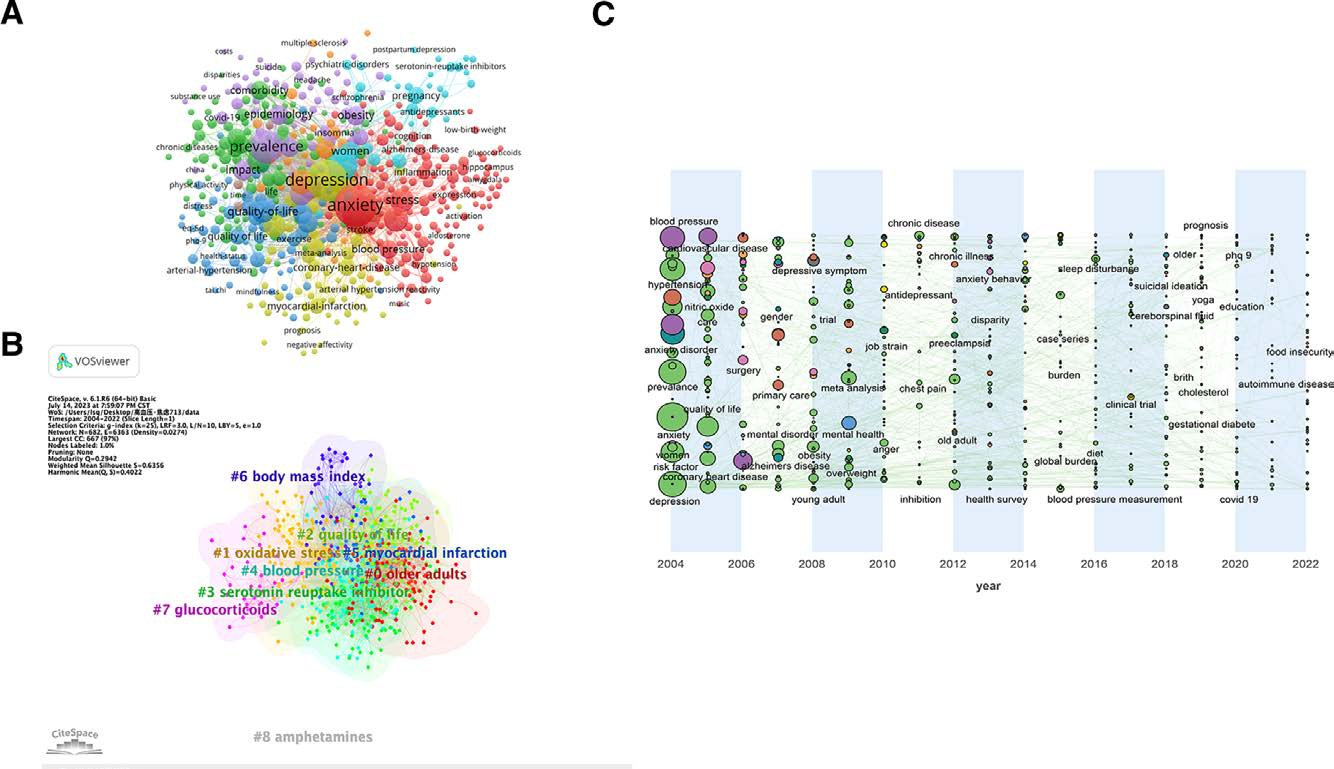
The analysis of keywords. (A) High-frequency keywords and its relationships in hypertension and anxiety research. (B) Keywords cluster analysis co-occurrence map. (C) Keyword time zone map. Size of the circle was positively correlated with attention.

Keyword analysis is a valuable approach to uncovering the fundamental content of literature, comprehending the developmental trajectory, identifying research focal points, gauging emerging trends, and predicting the future course of the subject matter. In this study, we focused on the “keyword” node and conducted a visual analysis of keyword co-occurrence as illustrated in Figure 6A. We conducted an analysis of the top 10 high-frequency keywords which were highly relevant to the topic in Table 6. Notably, the 3 most frequently occurring keywords were “anxiety” (1083), “depression” (939), and “hypertension” (840). Anxiety and hypertension as search terms are normal as frequent keywords in search results. Depression is closely related to anxiety, both being a type of adverse emotion, and the two often occur together.

**Table 6:** Top 10 high-frequency keywords and centrality indices in hypertension and anxiety research

No. 10	Occurrences	Total link relevance	keyword
1	1083	8877	anxiety
2	939	8042	depression
3	840	6850	hypertension
4	578	4806	prevalence
5	443	3636	risk
6	342	3069	association
7	290	2434	blood-pressure
8	285	2319	symptoms
9	268	2289	health
10	265	2143	stress

The LLR algorithm was employed to conduct cluster analysis of the keywords. In Figure 6B, we present the clustering of 7 keywords in WOS, which includes “#0 older adults,” “#1 oxidative stress,” “#2 quality of life,” “#3 serotonin reuptake inhibitor,” “#4 blood pressure,” “#5 myocardial infarction,” “#6 body mass index” and “#7 glucocorticoids.” Clusters #0, #1 can be classified as risk factors, clusters #2, #4, #6 can be classified as relevant evaluation index, clusters #3, #7 can be classified as treatments and clusters #5 is the disease it may causing.

It can be observed from the Figure 6C that the concerns have been changing. In earlier years, the circles corresponding to blood pressure, prevalence, and risk factors were large, indicating that these words received more attention. From 2004 to 2008, there was a change in the popularity of interest, and the research degree of quality of life, cardiovascular disease, and primary care of CHD increased. From 2008 to 2012, mate analysis, mental health, overweight, chronic diseases, anger became new research hotspot. From 2012 to 2022, the smaller circle means that the number of studies in this area is likely to be decrease. Research hotspot is mainly focused on the blood pressure measurement, prognosis, sleep disturbance, autoimmune diseases and prognosis.

### 3.5. Burst keyword

**Figure 7.**
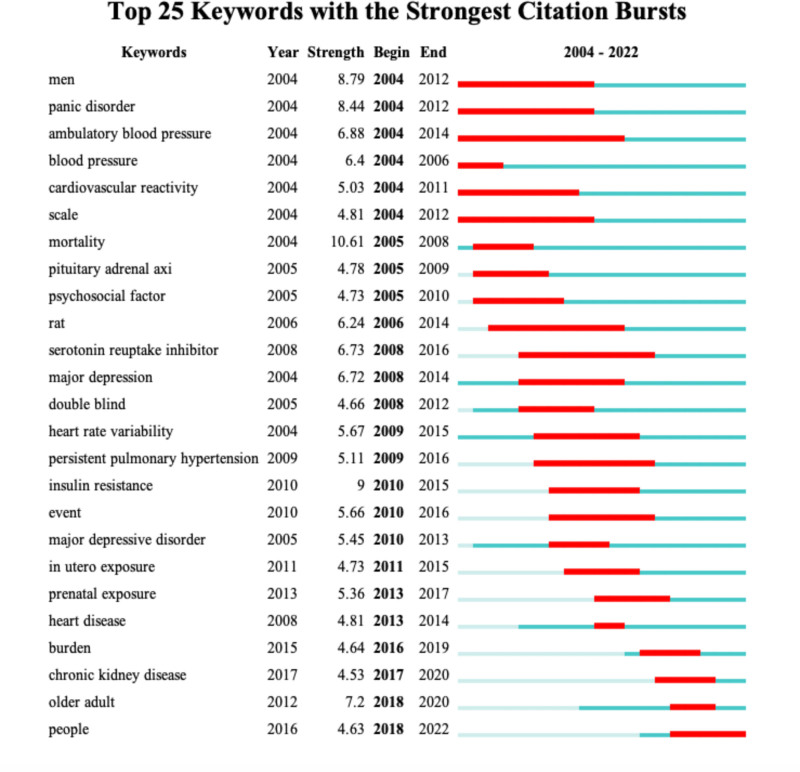
Top 25 keywords with the strongest citation bursts.

Burst keyword refers to the keywords that appear very frequently in the articles published in a short period of time, which can reflect the importance and attention of the keywords in the research field and predict the development trend. The bursting analysis of WOS literature data identified 25 words as Figure 7. After simple categorization, “men” is related to gender, “panic disorder,” “major depression” and “major depression disorder” related to mood, “ambulatory blood pressure,” “blood pressure,” “persistent blood pressure” and “blood pressure.” “Pressure” “blood pressure” “persistent pulmonary hypertension” is related to hypertension, “cardiovascular reactivity,” “heart rate variability,” “pituitary adrenal axi” and “insulin resistance” are related to physiologic and pathologic changes, “heart disease” and “chronic kidney disease” are related to diseases of various organs, “old adult” is related to age, “in utero exposure” and “prenatal exposure” and related to pregnancy and “serotonin reuptake inhibitor” is related to treatment. It is predicted that in the next few years, research will probably focus on risk factors for the disease as well as pathologic changes. As the results of clinical studies demonstrate that hypertension is associated with anxiety, subsequent research at the molecular level is likely to increase.

## 4. Discussion

### 4.1. Regular results

#### 4.1.1. Volume of publications

In this bibliometric analysis study, we found 3216 articles published from 2004 to 2022 in the WOS database. The overall publication numbers maintained a stable growth trend. Since 2018, the growth rate of the number of papers has accelerated, indicating that the field has received more and more attention.

#### 4.1.2. Organization

University of Toronto, Michigan State University and Harvard University which were the 3 most prolific agencies are all based in the US. Harvard university (3099), the university of Edinburgh (2491) and university of California (2202) are the 3 universities with the highest number of citations. Their respective number of publications are 40,18 and 37, indicating the high quality and credibility of the articles produced by these 3 institutions.

#### 4.1.3. Journal

The 3 journals with the largest amount of literature are Hypertension (1855), Lancet (1772), and Circulation (1750). Hypertension’s research area is peripheral vascular disease which is focus on the prevention and treatment of hypertension and related cardiovascular, metabolic and renal diseases. Lancet is the world’s leading comprehensive medical journal and has a wide range of global influence. It mainly in the field of medical internal medicine. The articles included in Circulation are mainly basic and clinical research in the field of cardiovascular disease, including myocardial infarction, angina, CHD, and so on. As three of the most highly published and cited journals in the field of hypertension and anxiety, they rank among the highest in terms of article quality, research hotspots and credibility.

#### 4.1.4. Country

The United States is more advanced in this area, ranking first in the number of publications, citations and greatest international cooperative efforts which far exceeding other countries. It shows that there is still a big gap between different countries. For a long time, the United States still has an important position and influence in this field. It is worth noting that most of them are European and American countries, and China is the only Asian country in the table.

#### 4.1.5. Keywords

With the diagrams in Figure 6, we can realize that the risk factor is presented as a large circle in the chart with the most frequency and attention in 2004. From 2004 to present, the risk factors of hypertension and anxiety disorders have been expanded by research. Gender, obesity, mental health, overweight, anger, sleep disturbance, cholesterol and gestational diabetes are associated with the risk factors for hypertension. Depression, job strain, and sleep disturbance are associated with risk factors for anxiety disorders. It is worth noting that in 2004, both men and women were the hotspots of attention, but the frequency of occurrence of the women counterpart was 131 times, which was much higher than that of men’s 51 times, and it can be concluded that women’s studies related to hypertension and anxiety were more concerned and more closely related.

Based on the results of Figure 6C, the diseases co-associated with hypertension and anxiety involve different disciplines, including cardiovascular, neurology and gynecology. Hypertension is associated with cardiovascular diseases, such as CHD. CHD is also associated with anxiety, which is often experienced by patients before and after the PCI surgery and bypass surgery. Anxiety is also a risk factor for CHD. In addition, it has been shown that hypertension is one of the important risk factors for Alzheimer’s disease, and antihypertensive treatment may have a protective effect against cognitive impairment and dementia. And there is a positive correlation between sudden anxiety and agitation in Alzheimer’s patients.^[21]^ Pre-eclampsia is a type of hypertensive disease in pregnancy, and patients with gestational diabetes have an increased risk of developing pre-eclampsia, and women in pregnancy should have reasonable glycemic control. In addition, pregnant women are prone to anxiety,^[22]^ which is related to both age and education.

In 2005, quality of life became the research topic of highest interest, corresponding to the largest circle in the chart. The latest hypertension guidelines change lifestyle interventions from a 7-part program to an 8-part program that includes reducing sodium intake, increasing potassium intake, eating a sensible diet, controlling body weight, not smoking, limiting alcohol consumption, increasing physical activity, psychological balance, and managing sleep, with the addition of “managing sleep” as a measure. Sleep disorders and hypertension interact with each other.^[23]^ High blood pressure may cause dizziness and headaches that affect sleep quality, while poor sleep may cause blood pressure to fluctuate and lead to elevated blood pressure. Dietary management has always been important in the management of hypertension, and 4 of the 8 components are related to dietary management. People with the disease should pay attention to the intake of sodium and potassium on the one hand, and the control of blood lipids on the other. Heavy alcohol consumption and intake of high cholesterol substances can cause an increase in blood lipids and trigger CHD, which is typically characterized by chest pain. In addition, anxiety and the quality of life also interact with each other, mainly in terms of sleep. Anxiety is often accompanied by insomnia, which in turn exacerbates anxiety. In addition, job strain may affect quality of life by causing abnormalities in mental and physical health, and is a contributing factor to hypertension and anxiety. The main manifestations are anger, sleep disturbance, suicidal ideation. For health abnormalities, health surveys, blood pressure measurements, and phq 9 scales are gaining importance. These are mainly used for confirmatory diagnosis of diseases. Compared to the early years when the focus of research was on the disease itself, with the development of technology, the focus of research has shifted first to the symptoms of the disease, and then to the precise diagnosis and prognosis of the disease, which reflects the increasing demand for quality of life.

Primary care is characterized by hierarchical diagnosis and treatment, and has received great attention since 2007. Community medicine is the most common form of primary care, in which community doctors are connected to residents as family doctors, keeping a close eye on their health and helping them to “prevent and treat chronic diseases.” Hypertension is one of the diseases that are managed in the community. Family doctors monitor the blood pressure of hypertensive patients, so that the management of their lives can be more refined and individualized. The prevention and treatment of anxiety disorders in primary care has also been emphasized and continuously optimized, and the methods of identifying and managing anxiety disorders are continuously updated.^[24–26]^

In recent years, attention has been focused on diet, exercise, education and prognosis, and people are more concerned about maintaining and achieving a healthy state through a healthy lifestyle. This is also a sign of people’s growing concern for quality of life.

### 4.2. Risk factor

Risk factors for hypertension and anxiety are important. The effect of risk factors on the disease was repeatedly mentioned in the results of the keyword analysis in the previous section, mainly including gender, age, obesity, depression and panic disorder, pregnancy induced hypertension, complication, treatment and other. So here the risk factors are discussed and analyzed.

#### 4.2.1. Gender is both associated with hypertension and anxiety

The prevalence is generally higher in men than in women. However, the increase in prevalence after menopause is greater in females than in males, and the prevalence is elevated or even exceeds that of males.^[27,28]^ Men, urban residence, abdominal obesity and hypercholesterolemia significant factors of hypertension in young adults.^[28]^ The high prevalence in males may be attributed to poor lifestyle habits such as smoking and alcohol consumption, social stress, and the role of androgens. Hyperandrogenism leads to abnormalities in lipid metabolism^[29,30]^ and activation of the RAAS system^[31]^ leading to the development of hypertension. The high prevalence in menopausal women may be related to sex hormones, renin-angiotensin-aldosterone, sympathetic nervous system and atherosclerosis. During menopause, the prevalence of hypertension and its complications increases dramatically in women^[32]^ and may be related to atherogenic dyslipidemia, central obesity and insulin resistance,^[33]^ autophagy, decreased ER alpha expression, and excessive damage to the ascending aorta.^[34]^ Estrogen can prevent atherosclerosis by upregulating ER alpha expression, which in turn induces autophagy, reduces inflammation and pyroptosis,^[34]^ and can increase nitric oxide bioavailability, reduce oxidative stress and inflammation, and improve vascular endothelial function thereby regulating blood pressure.^[35]^

Arcand, M et al conducted a scale analysis of 108 students (50 males) and 151 workers (75 males) aged 18-65 years and found that the stronger the masculinity, the lower the symptoms of anxiety, and the higher the feminization, the higher the anxiety.^[36]^ A survey involving 316 people carried out in the Nepali community found that 36.9% of females identified anxiety symptoms through scale assessment under the influence of stressful social events and social support, which was higher than that of males, which was 20.4%.^[37]^ The occurrence of anxiety was also found to be higher in girls than boys in math.^[38]^

Most of the current studies support that females are more prone to anxiety, but due to the large number of factors that lead to anxiety, the lack of related animal experiments and mechanism studies and a number of studies crystallize anxiety, such as math anxiety, social anxiety, etc., so it is not yet possible to conclude that anxiety is necessarily correlated with gender. More experimental data is needed to support the proof of the conclusion.

#### 4.2.2. Age strongly associated with hypertension and anxiety

As the human body ages, the blood vessel wall gradually loses its elasticity, and at the same time the intima is damaged to a certain extent, leading to an increase in vascular resistance, which in turn triggers hypertension. The mechanism of action may be related to atherosclerosis, endothelial damage, and decreased kidney function.^[39–41]^ With age, the arterial wall gradually becomes hardened and loses elasticity, the inner diameter of the blood vessel narrows, and the cardiac load increases.^[39]^ At the same time, the structure of the intima may change,^[40]^ and it may be damaged by various factors, such as hyperglycemia, hyperlipidemia, inflammation, and so on. These injuries cause inflammatory reactions and plaque formation in the endothelium, which in turn leads to thickening of the vessel wall and interferes with blood flow. The decline in renal function, which leads to fluid retention and decreased sodium excretion, which in turn leads to an increase in blood pressure.^[40]^

There is also a relationship between anxiety and age, with anxiety occurring differently at different ages. Adolescent anxiety is mainly characterized by separation anxiety, social anxiety, fear of failure and criticism, and anxiety due to fear of death and danger.^[41]^ Pathologically, hypertension in adolescents is strongly associated with obesity^[42]^ in addition to family history.^[43,44]^ Anxiety was found to be related to gender, marital status, health, income level, and quality of life in a survey that included 3159 older adults.^[45]^

#### 4.2.3. Obesity has an effect on both hypertension and anxiety

Insulin resistance plays an important role in the pathogenesis of type 2 diabetes mellitus and is mostly seen in obese patients. Obesity is also a causative factor of insulin resistance.^[46]^ The mechanisms of action are as follows:

1. Activation of the renin-angiotensin-aldosterone system: Adipose tissue contributes to increased circulating levels of AngII and aldosterone, which not only causes sodium retention and sympathetic excitation, but also may impair microvascular function and lead to atherosclerosis, ultimately leading to high blood pressure.^[47]^
2. Neuroendocrine disorders: Obesity is associated with disorders of the autonomic nervous system,^[48]^ and sympathetic hyperexcitability may lead to vasoconstriction and cause hypertension. Increased renal sympathetic nerve activity increases sodium reabsorption in obese patients, which can also lead to the development of hypertension.^[49]^
3. Inflammatory response: Inflammation leads to endothelial cell damage and inflammatory response in the vascular wall, which affects vascular function and makes blood pressure rise.^[50]^ In addition, healthy adipose tissue secretes many vasoactive adipokines and anti-inflammatory cytokines that have both anticontractile and pro-contractile effects on the vascular system. Changes in the secretion profile in obese patients may result in the loss of anticontractile effects, leading to increased vascular tone, increased vascular stiffness, and increased RAAS activity, all of which may contribute to hypertension.^[51]^

Obesity is strongly associated with anxiety. In an interview with obese women, 38.2% of them showed symptoms of anxiety.^[52]^ An animal experiment found that anxiety-like behaviors observed in male obesity-prone rats were positively correlated with obesity.^[53]^ The mechanism may be that obesity leads to metabolic disorders that disrupt neurological function, which in turn leads to anxiety.^[54]^

#### 4.2.4. Depression and panic disorder often co-occur with hypertension and anxiety

Hypertension and depression have significant co-occurrence.^[55]^ Moderate depression has a significant synergistic effect on hypertension.^[23]^ The rates of depression and anxiety symptoms in hypertensive patients were 27.2% and 32.7%.^[56]^ In another study of 30,434 individuals, depression was found to be significantly associated with an increased risk of hypertension compared to those without depression.^[23]^ There is a positive correlation between panic disorder and lifetime development of hypertension and hypertension and the subsequent development of panic disorder.^[57]^

The prevalence of panic disorder is tripled in patients with primary hypertension. Elevated plasma cortisol, recurrent discharges of individual sympathetic nerve fibers usually within a single cardiac cycle, and elevated tissue nerve growth factor are seen in both panic disorder and essential hypertension, which may be related to pathogenesis.^[58]^

There is an association between depression, panic disorder and anxiety. Out of 1346 patients with major depressive disorder, 286 (21.2%) were found to present with comorbid anxiety disorders, 10.8% with generalized anxiety disorder, and 83% with panic disorder.^[59]^ One study identified comorbid anxiety disorders as a unique risk factor for panic disorder.^[60]^

#### 4.2.5. Depending on the individual, high blood pressure may occur during pregnancy and there is a tendency to experience anxiety

The pathogenesis of gestational hypertension may be related to the following:

1. Abnormal vascular function: Umbilical cord plasma exosomes (PE-uexo) from women with preeclampsia disrupt the normal function of vascular endothelial cells,^[61]^ and phagocytosis of necrotic trophoblast cell debris by endothelial cells can cause endothelial cell dysfunction,^[62]^ which can lead to hypertension.
2. Immune system dysregulation: Abnormalities of the immune system and increased inflammatory response may affect vascular function and lead to increased blood pressure.^[63]^ In addition, inflammation and oxidative stress are prominent features of preeclampsia (PE), and in utero exposure to this inflammatory and oxidative environment may affect blood pressure and the neurodevelopment of the offspring.^[64]^
3. Increased blood flow: The maternal cardiovascular system undergoes major changes during pregnancy. To meet the nutrient and oxygen requirements for normal fetal development, blood flow in the uterine arteries (UtA) required for uteroplacental circulation increases,^[65]^ which may cause an increase in blood pressure.
4. Thrombosis and platelet aggregation: Pregnant women with hyperemesis gravidarum are in a hypercoagulable state and are at higher risk of thrombosis and secondary hyperfibrinolysis.^[66]^ Thrombosis and platelet aggregation may cause vascular stenosis, which can lead to elevated blood pressure.

Depression, stress and anxiety scores were higher in patients with gestational hypertension than in those who did not suffer from hypertension.^[11]^

### 4.3. Complication

#### 4.3.1. CHD correlates with anxiety and interacts with hypertension

Heart disease includes many kinds of cardiovascular diseases, among which CHD is more closely associated with hypertension, so the relationship between CHD, hypertension and anxiety is discussed here.

CHD and hypertension are closely related.^[67]^ Of the 14,180 patients who underwent PCI 11,066 (78.0%) patients had hypertension. Hypertension is one of the risk factors^[68]^ as well as causative factors^[69]^ for CHD and aggravate the progression of CHD. Chronic hypertension leads to cardiac hypertrophy and insufficient blood supply to the myocardium, increasing the risk of coronary atherosclerosis. Patients with coronary artery disease may develop narrowed or blocked coronary arteries, which can limit the blood supply to the heart and also cause a sharp rise in blood pressure, which can ultimately increase the prevalence of coronary artery disease and increase the risk of death.

CHD and anxiety are also correlated.^[70,71]^ Some studies have noted that the prevalence of CHD in patients with anxiety disorders is increased by about 3 times. The increased prevalence of CHD among depressed patients is mainly due to comorbid anxiety.^[71]^ However, it has also been shown that both depression and anxiety are significantly associated with coronary artery disease, but the correlation between depression and coronary artery disease is more significant compared to anxiety.^[72]^ Patients with coronary artery disease are more prone to anxiety as well as depression and other adverse emotions before^[73,74]^ and after PCI surgery.^[75,76]^

#### 4.3.2. Hypertensive nephropathy in chronic kidney disease are associated with hypertension and anxiety

Chronic kidney disease (CKD) mainly includes various types of nephritis, nephrotic syndrome, membranous nephropathy, hypertensive renal damage, diabetic nephropathy, and so on. Among them, hypertensive nephropathy is one of the complications of hypertension, which is closely related to this subject word “hypertension,” and hypertension is a risk factor as well as a complication of CKD,^[77]^ and the two kinds of malignant influence on each other,^[78]^ which may be one of the reasons why it has become an burst keyword. The mechanism may be related to immune dysregulation, metabolic disorders and sympathetic activation.^[79]^ CKD is also associated with high anxiety.^[80]^ In a questionnaire survey of patients with chronic kidney disease, more than half of the participants suffered from depression (58.3%) and anxiety (50.5%).^[81]^

### 4.4. Serotonin reuptake inhibitor is a medication used to treat psychiatric disorders for which there is insufficient evidence for the treatment of hypertension

5 -Serotonin reuptake inhibitor (SRI) appeared in the keyword clustering analysis, and the burst keywords. 5-Hydroxytryptamine (5-HT) has an arteriolar constricting effect and is a mitogen of vascular smooth muscle cells, which promotes the contraction of smooth muscle growth, both of which contribute to hypertension.^[82]^ But SRI is a class of antidepressant drugs used in the treatment of depression and obsessive-compulsive disorder by inhibiting the reuptake of 5-HT through selective action on the presynaptic membrane,^[83,84]^ and its mechanism of action may be related to the absolute reduction of platelet serotonin reuptake.^[85]^ However, the exact mechanism of action is not clear. There is less literature on SRI for the treatment of hypertension, and some researchers have noted an association between SRI and arterial hypertension.^[86]^ Possibly related to the search terms, a larger portion of the literature searched was related to pulmonary hypertension as well as selective serotonin-norepinephrine reuptake inhibitors. SRI has been shown to reduce pulmonary hypertension by inhibiting pulmonary vascular remodeling and reducing inflammation.^[87–89]^ There is also experimental evidence that serotonin-norepinephrine reuptake inhibitors is involved in the control of inflammation, cognitive function, motivational behavior, and neuronal degeneration, and can be effective in alleviating the harm caused by renal vascular hypertension.^[90]^ Furthermore, several studies on SRI-use during pregnancy that point to the possibility of affecting placental or fetal serotonin levels even when maternal antidepressants are taken at therapeutic doses,^[91]^ which may lead to persistent pulmonary hypertension and congenital heart disease in newborns.^[92]^

### 4.5. Other

#### 4.5.1. Pituitary adrenal axi plays a role in both hypertension and anxiety pathogenesis

The hypothalamic-pituitary-adrenal axis, which includes 3 main components: the hypothalamus, the pituitary gland, and the adrenal cortex, plays an important role in the onset and progression of hypertension. The activation of the RAAS system is closely related to the development of hypertension.^[93]^ The anterior pituitary releases corticotropin-releasing hormone, which stimulates the activation of the hypothalamic-pituitary-adrenal axis, causing the adrenal glands to produce adrenocorticotropic hormones,^[94]^ such as cortisol. Cortisol increases renal tubular reabsorption of sodium ions and indirectly contributes to the activation of the renin-angiotensin system which leads to vasoconstriction and an increase in body fluid volume, thereby elevating blood pressure.

In addition, the pituitary gonadal axis and pituitary thyroid axis are also associated with hypertension. Abnormalities of the pituitary gonadal axis interfere with the normal secretion of sex hormones and have been discussed in the context of the relationship between gender and hypertension. The relationship between elevated blood pressure and overt thyroid dysfunction has been well studied and confirmed, and atherosclerotic changes due to lipid abnormalities caused by thyroid dysfunction also affect the vascular system and may lead to elevated blood pressure.^[95]^ There is also a significant correlation between thyroid stimulating hormone levels and arterial blood pressure and hypertension within the normal range.^[96]^

The pathway of stress and anxiety also involves the HPA axis.^[97]^ The mechanism of action may be that hyperactivation of the HPA axis triggers multiple neuroendocrine responses that promote anxiety-like behaviors.^[98]^

#### 4.5.2. Pulmonary hypertension is not significantly related to the study topic of this paper

There is no necessary link between pulmonary hypertension and hypertension and anxiety. Pulmonary hypertension is also a disease of increased intravascular pressure, but it is different from conventional systemic hypertension. Severe pulmonary hypertension can lead to impaired heart function, develop into pulmonary heart disease, lead to poor blood circulation, the formation of hypertension often associated with right-heart failure, the patient often accompanied by unavoidable anxiety. The high frequency of pulmonary hypertension may be related to the research term “hypertension” itself, and we want to focus on hypertension, not pulmonary hypertension.

### 4.6. The relationship between hypertension and anxiety

There is a bridge between hypertension and anxiety, and the mechanism of its occurrence may be related to activation of the renin-angiotensin system, thrombosis, inflammation development, increased ion reabsorption, aortic atherosclerosis, and endothelial dysfunction vascular reactivity. The role of the RAS in hypertension and mood disorders is now well recognized.^[99,100]^ RAS activation leads to an increase in blood pressure by inducing local coronary artery constriction, increasing sympathetic excitability, and promoting sustained aldosterone release. At the same time, RAS also contributes to anxiety onset,^[101]^ and its mechanism of action may be induction of neuroinflammation and oxidative stress.^[99]^ Elevated levels of IL-6^[102]^ and IL-17 have been observed in patients with anxiety disorders.^[103,104]^ IL-6 receptors are involved in and promote thrombosis, stimulate chronic inflammation,^[102]^ promote Na + reabsorption^[105]^ and activation of the RAS system,^[106]^ leading to hypertension; IL-17 promotes inflammation^[107]^ and activates RhoA/Rho-kinase,^[108]^ leading to endothelial dysfunction and hypertension. In addition, ROS accumulation leads to an imbalance in synaptic function, stimulating inflammation, aortic atherosclerosis, RAS dysfunction, endothelial dysfunction, vascular hyperresponsiveness and structure remodeling, which are directly involved in the development of hypertension and anxiety.^[106]^ A search in WOS revealed a relatively limited number of articles on how hypertension triggers anxiety, so it will not be discussed here.

## 5. Conclusion

In this study, the bibliometric analysis provided the current status of research in the field of hypertension and anxiety as well as research trends from 2004 to 2022. There is an upward trend in the number of publications in the field with increasing attention and a wide range of studies. The results of the literature analyses show a link between hypertension and anxiety, which interact with each other, often with a positive correlation. According to the visualization results and literature analysis, the common influences of hypertension and anxiety are age, gender, obesity, depression, panic disorder, pregnancy, CHD and chronic kidney disease. Research hotspots in recent years have focused on population aging and complication. Current research on hypertension is relatively complete, but there are few studies on hypertension and anxiety, and the mechanism of their occurrence is still incomplete. Most of the literature has focused on clinical studies, with fewer studies at the molecular level. Future research hotspots are likely continue to focus on influencing factors, clinical research and prognosis. Researchers can explore the mechanisms of their interactions and interventions in the future to provide a more comprehensive perspective to better improve cure rates and promote further development in this area.

## Acknowledgments

We extend our heartfelt gratitude to the Shuai Shi for the unwavering support and great contribution to this project.

## Author contributions

**Conceptualization:** Hai-Yi Liang.

**Data curation:** Hai-Yi Liang

**Formal analysis:** Shu-Han Zhao.

**Funding acquisition:** Xin-Yu Ji.

**Investigation:** Shu-Han Zhao.

**Methodology:** Siqi Liu, Fu-Yi Yang.

**Resources:** Xin-Yu Ji, Fu-Yi Yang.

**Software:** Siqi Liu, Fu-Yi Yang.

**Supervision:** Shuai Shi, Yang Tang.

**Validation:** Shuai Shi, Yang Tang.

**Visualization:** Xin-Yu Ji.

**Writing – original draft:** Siqi Liu.

**Writing – review & editing:** Siqi Liu.
